# Inequalities in recovery or methodological artefact? A comparison of models across physical and mental health functioning

**DOI:** 10.1016/j.ssmph.2022.101067

**Published:** 2022-03-05

**Authors:** Salmela Jatta, Brunton-Smith Ian, Meadows Robert

**Affiliations:** a Department of Public Health, University of Helsinki, Tukholmankatu 8 B, PO Box 20, 00014, Helsinki, Finland; b Department of Sociology, Elizabeth Fry Building (AD), University of Surrey, Guildford, Surrey, GU2 7XH, UK

**Keywords:** Health inequalities, Recovery, Socioeconomic inequalities, Trajectory modelling, United Kingdom, AMI, Acute myocardial infarction, APP, Average posterior probability, BIC, Bayesian information criterion, CI, Confidence interval, GBTM, Group-based trajectory modelling, GCSE, General Certificate of Secondary Education, MCS, Mental component summary, OCC, Odds of correct classification, OR, Odds ratio, PCS, Physical component summary, SES, Socioeconomic status, SF-12, 12-Item Short Form Survey, SF-36, 36-Item Short Form Survey

## Abstract

Considerable attention has been paid to inequalities in health. More recently, focus has also turned to inequalities in ‘recovery’; with research, for example, suggesting that lower grade of employment is strongly associated with slower recovery from both poor physical and poor mental health. However, this research has tended to operationalise recovery as ‘return to baseline’, and we know less about patterns and predictors when recovery is situated as a ‘process’. This paper seeks to address this gap. Drawing on data from the UK Household Longitudinal Study, we operationalise recovery as both an ‘outcome’ and as a ‘process’ and compare patterns and predictors across the two models. Our analysis demonstrates that the determinants of recovery from poor health, measured by the SF-12, are robust, regardless of whether recovery is operationalised as an outcome or as a process. For example, being employed and having a higher degree were found to increase the odds of recovery both from poor physical and mental health functioning, when recovery was operationalised as an outcome. These variables were also important in distinguishing health functioning trajectories following a poor health episode. At one and the same time, our analysis does suggest that understandings of inequalities in recovery will depend in part on how we define it. When recovery is operationalised as a simple transition from poor health state to good, it loses sight of the fact that there may be inequalities (i) within a ‘poor health’ state, (ii) in how individuals are able to step into the path of recovery, and (iii) in whether health states are maintained over time. We therefore need to remain alert to the additional nuance in understanding which comes from situating recovery as a process; as well as possible methodological artefacts in population research which come from how recovery is operationalised.

## Highlights

• There is no consensus on what recovery is and how it should be operationalised.
• Understanding of inequalities in recovery across health conditions remains scarce.
• We operationalised recovery both as an outcome and as a process.
• We found robust inequalities in recovery across the two approaches.
• Considering recovery as a process revealed more nuanced patterns in inequalities.

## 1 Introduction

Health inequalities continue to grow wider (Marmot, 2020) and there remain significant differences in health by axes of social inequality such as gender, race/ethnicity, income and neighbourhood deprivation (Bambra, 2021). For example, low socioeconomic status (SES) continues to be one of the strongest predictors of morbidity and mortality (Stringhini et al., 2017; Young, 2004). Women also outlive men in nearly all countries of the world (Annandale, 2014) and rates of disease and death are elevated for historically marginalised groups (Williams et al., 2019). African Americans have a lower life expectancy at age 25 than do Whites, and this persists at every level of education and income (Williams et al., 2019).

Research has also pointed towards disparities and variation in ‘recovery’ from poor physical and mental health. Drawing on data from 229 people discharged from hospital after acute myocardial infarction (AMI), Lacey and Walters (2003) found women showing poorer improvement than men and those without educational qualifications showing poorer improvement in vitality and pain experience. As well, Dreyer et al. (2015) measured health status at three time points after AMI and found women having significantly lower health status scores than men at each assessment. Beyond AMI, Chen et al. (2015) found socioeconomic inequalities in recovery after stroke, where those in the second, third and fourth index of multiple deprivation experienced worse stroke-related recovery. Dowrick et al. (2011) found that recovery from depression was associated with socioeconomic diversity and homeownership. Furthermore, Putman's et al. (2007) study illustrated SES playing an important role in functional recovery after stroke, while the contributions of educational and equivalent income levels varied depending on the stage in recovery process. However, Connor et al. (2001) found that rate of recovery of aphasia was the same regardless of educational and occupational status.

Despite speaking to a diverse range of illnesses and conditions, the literature on inequalities and determinants of recovery appears united by two key issues. First, whilst the term recovery is frequently used (or alluded to), it is not always defined. It is often left implicit that it is something akin to an ‘outcome’ or ‘return to baseline’. Conceptually, this risks negating the numerous complexities and ambiguities surrounding recovery and mental/physical health. For example, whilst the clinical literature often situates mental health recovery as the amelioration of symptoms so that a person can resume activities within what is considered a normal range (Meadows et al., 2020), policy documents such as ‘No Health without mental health’ (2011), follow Anthony's definition (1993). Here mental health recovery is situated as “a deeply personal, unique process of changing one's attitudes, values, feelings, goals, skills and/or roles. It is a way of living a satisfying, hopeful and contributing life, even with limitations caused by the illness.” (p. 527). A similar situation can be seen with recovery from acute physical illness or serious injury. Chen et al. (2015) use differences in functional impairment as a proxy of recovery after stroke, whilst for the sociologist Frank (2013) a core part of recovery is what you learn about the life you are regaining. Even a seemingly simple concept such as ‘survivorship’ has been shown to lack a consistent operational definition (Khan et al., 2012). This lack of conceptual clarity may also serve to make findings invisible. Despite work on determinants of recovery, it continues to be suggested that dominant discourses individualise what are social problems (Harper & Speed, 2014), and the literature has remained ‘silent’ about the socio-structural conditions of living (Karadzhov, 2021). Ambiguities may be worsening patients' conditions (Friedman, 2021) and studies on determinants need to think beyond ‘recovery as an outcome’ models.

Second, it is unclear whether known patterns and predictors of mental/physical health recovery are dependent on the way in which recovery is operationalised in analysis. This resonates with early discussions of inequalities in health more generally. Over 40 years ago the Black Committee proposed four types of explanation of class differences in health (artefact, social selection, cultural/behavioural and materialist) (Davey et al.,1994). A ‘hard’ version of the artefact explanation suggests that there is no relation between class and mortality and that any observed relationship is purely an artefact of measurement. A ‘soft’ version suggests that the magnitude of observed class gradients will depend on measurement of both class and health (Macintyre, 1997; Bloor et al., 1987). For Davey et al. (1994), artefactual factors mean that we are underestimating socio-economic differentials. It has been suggested that choice of survival measure affects the estimation of social class differences (Dickman et al., 1998), and studies have considered artefacts as a possible explanation for class differences in cancer patients' survival (Auvinen & Karjalainen, 1997). Moving beyond ‘recovery as an outcome’ models will also help understand the role methodological artefact plays in possible explanations for difference.

This study looks to engage directly with these issues by using large-scale, representative data to operationalise recovery as both an ‘outcome’ and as a ‘process’ and to compare patterns and predictors across the two models. We ask: (i) Can multiple, unique, trajectories of recovery be identified? (ii) How do age, gender, ethnicity, marital status, education and employment status relate to these trajectories of recovery? (iii) How do findings from these process models compare with models that explore odds of moving from a poor health state to a good health state (recovery as an outcome)? This dualism of ‘process/outcome’ reflects the fact that complexities surrounding recovery are often summarised in terms of a series of either/ors. For Davidson and Roe (2007) it is ‘recovery from’ versus ‘recovery in’, for Slade et al. (2008) it is a ‘clinical recovery’ versus a ‘personal recovery’ and for Liberman and Kopelowicz (2005) it is ‘recovery as an outcome’ versus ‘recovery as a process’.

We prioritise self-report measures over clinical measures within this study. Whereas existing population work on inequalities has tended to err towards ‘from’/’clinical’/’outcome’, qualitative work has emphasised the everyday importance of recovery as a process; something which is more personal, unique, and complex (Godfrey & Townsend, 2008; Southby et al., 2021). Following Tanaka et al. (2018), we also look at general measures of physical and mental health, rather than specific illnesses or diagnosis. Drawing on the Whitehall II cohort study and using the physical and mental component scores of the Short Form 36 (SF-36) questionnaire, Tanaka et al. (2018) found that lower employment grades were strongly associated with lower odds of recovery from poor physical and, in particular, mental health, after 5-year periods. However, these findings rely again on ‘recovery as an outcome’ approach, thus leaving a question whether the findings would be different if recovery is operationalised as a process.

## 2 Materials and measures

We draw on data from the UK Household Longitudinal Study, which is designed to be representative of the UK population, including all ages, areas of the UK and educational and social backgrounds (McFall & Garrington, 2011). Approximately 40,000 households were recruited to Wave 1 in 2009–2010 (McFall & Garrington, 2011), and respondents have been interviewed annually since. The total number of respondents varies between 36,055 and 54,569 in Waves 1–9, and the age distribution remains similar across the waves (University of Essex, Institute for Social and Economic, 2019). The survey includes questions on a wide range of topics, including health and disability, sleep, partnership history, caring, family networks, employment and relationship quality.

**Fig. 1:**
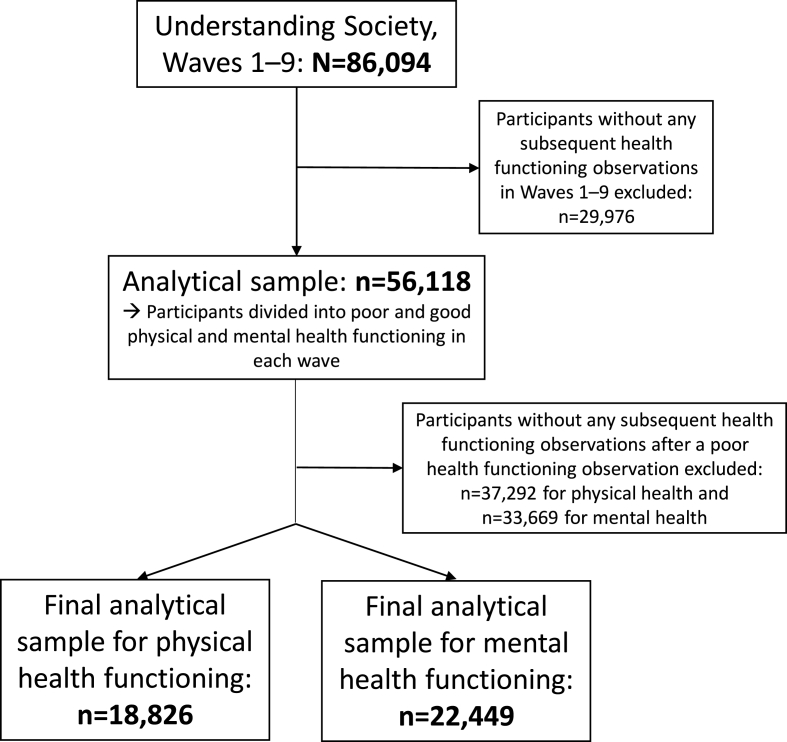
Flowchart: inclusion criteria for the final analytical study samples.

Fig. 1 details our final analytical sample. The inclusion criteria were based on available data on participants’ health functioning from Wave 1 to 9. Health functioning (i.e., the outcome variable) was measured using the physical and mental health component summary (PCS and MCS) scores from the 12-Item Short Form Survey (SF-12) (Ware et al., 1996). The SF-12 is a shortened form of the SF-36, a widely used general health questionnaire. The SF-12 consists of 12 chosen items from the SF-36 and captures eight sub-scales, similarly to the SF-36: 1) physical functioning, 2) social functioning, 3) role limitations due to physical problems, 4) role limitations due to emotional problems, 5) mental health, 6) energy/vitality, 7) bodily pain and 8) general health perceptions. In the aggregated weighted summary scores of PCS and MCS, the mean is set to 50 and standard deviation to 10 in the general population, and higher scores indicate better health (Ware et al., 1996). The scores received from the SF-12 closely correspond to the scores from the SF-36 (Gandek et al., 1998; Jenkinson et al., 1997).

The initial analytical sample (n = 56,118) comprised those respondents who were included in Wave 1 and had at least one subsequent health functioning variable in Waves 2 to 9 (see Table S1). As our interest is primarily in those who begin with poor health, only participants who had at least one subsequent health observation after a poor health observation were included in the final analyses: that is, 18,826 participants for physical health analyses and 22,449 participants for mental health (Fig. 1). We defined poor physical/mental health as belonging to the lowest component score quintile (20%) among the analytical sample, similarly to previous studies (e.g., Raymakers et al., 2018; Tanaka et al., 2018; Ul-Haq et al., 2014), and this was calculated separately at each wave. The upper score limits of poor health were 40.8–41.4 for physical health and 40.4–44.0 for mental health. Participants in the analytical sample of mental health were more often younger, never married, higher educated, employed and full-time students, compared to participants in the analytical sample of physical health (Table 1). Being female, belonging to a White ethnic group and being in partnership represented a majority of the study participants.

**Table 1:** Description of the study population at the time of first poor health observation. Participants with at least one subsequent health observation after a poor health functioning observation during Waves 1–9 are included.

	Physical health (n = 18,826)	Mental health (n = 22,449)
Age (n, %)		
39 years old/younger	4180 (22.2)	9858 (43.9)
40–64 years old	8447 (44.9)	9418 (42.0)
65 years old/older	6199 (32.9)	3173 (14.1)
**Gender (n, %)**		
Female	11,123 (59.1)	13,961 (62.2)
Male	7703 (40.9)	8486 (37.8)
**Ethnicity (n, %)**		
White	15,876 (84.4)	18,533 (82.7)
Mixed	284 (1.5)	490 (2.2)
Asian/Asian British	1826 (9.7)	2295 (10.2)
Black/Black British	684 (3.6)	938 (4.2)
Other ethnic group	136 (0.7)	162 (0.7)
**Marital status (n, %)**		
Partnership	11,815 (62.8)	12,780 (57.0)
Widowed	1961 (10.4)	1182 (5.3)
Divorced/separated	2056 (10.9)	2245 (10.0)
Never married	2980 (15.8)	6219 (27.7)
**Educational attainment (n, %)**		
Higher degree	3883 (25.2)	5837 (30.8)
A-level/equivalent	1193 (7.7)	2281 (12.0)
GCSE/equivalent	3915 (25.4)	5564 (29.4)
Other qualification	595 (3.9)	394 (2.1)
No qualification	5851 (37.9)	4862 (25.7)
**Employment status (n, %)**		
Employed	6920 (36.8)	11,145 (49.7)
Unemployed	1040 (5.5)	1797 (8.0)
Retired	6819 (36.2)	3666 (16.3)
Family care	1476 (7.8)	1846 (8.2)
Full-time student	617 (3.3)	2159 (9.6)
Other	1951 (10.4)	1833 (8.2)

The predictors of recovery included six indicators of participants' demographic and socioeconomic characteristics: age, gender, ethnicity, marital status, educational attainment and employment status. Age, which ranged from age 16 to age 101 in Waves 1–9, was classified into three groups: 39 years old or younger, 40–64 years old and 65 years old or older. Gender included two groups: female and male. Ethnicity data distinguishes between five aggregated ethnic groups: White (e.g., English, Scottish, Irish or Gypsy), Mixed (e.g., ‘White and Black Caribbean’ or ‘White and Asian’), Asian or Asian British (e.g., Indian, Pakistani or Chinese), Black or Black British (e.g., Caribbean or African) and ‘Other’ ethnic group (i.e., Arab or any other ethnic minority groups that was not included within previous groups). Marital status was coded into four groups: partnership (married, civil partner or living as couple), widowed (including surviving civil partner), divorced/separated (including from civil partner) and never married. Highest education qualification was recoded into five groups: degree or above, A-level or equivalent, GCSE or equivalent, other qualification and no qualification. Employment status was recoded into employed, unemployed, retired, family care (including maternity leave), full-time student and ‘other’ (including, for example, long-term sickness and disability or government training scheme). Gender and ethnicity were considered as time-invariant variables, whereas age, marital status, educational attainment and employment status were considered as time-varying variables.

## 3 Methods

### 3.1 Model 1: approximating recovery as an ‘outcome’

Building on the approach adopted by Tanaka et al. (2018), we defined recovery as a transition from poor health state to good health state (i.e., belonging to the four highest component score quintiles [80%] in the initial analytical sample) between two subsequent waves. Thus, the outcome was binary: recovered (poor-good) and non-recovered (poor-poor). Overall, recovery from poor physical health was less common than recovery from poor mental health: a third of participants who reported poor physical health functioning in any of the Waves 1–8 recovered, whereas almost half of those who reported poor mental health functioning recovered (Table S1). To estimate inequalities in recovery, we ran separate binary logistic regression models for each of the eight periods (i.e., two subsequent waves). Time-varying predictors (i.e., age, marital status, education and employment status) were measured at the beginning of a period. Since the inequalities in recovery were parallel between the periods (Table S2), we merged the periods using multilevel logistic regression analysis. There, periods were nested within individuals, and consequently, multiple observations of recovery across periods on the same individual were allowed.

### 3.2 Model 2: approximating recovery as a ‘process’

Reflecting the idea that individuals have varying pathways in how they recover from poor health over time, we identified latent ‘recovery trajectories’ among the study population. Participants' first poor health observation during Waves 1–8 was considered as a starting point of the trajectories. Thus, 1–8 subsequent health observations after a first poor health observation constituted participants' recovery trajectories. The trajectories were based on raw (continuous) PCS and MCS scores, and we used group-based trajectory modelling (GBTM) (Nagin, 2005; Nagin & Odgers, 2010) for identifying the trajectory groups. GBTM is a finite mixture modelling application, which assumes that the study population consists of latent clusters of individuals who have somewhat homogeneous developmental trajectories in the outcome of interest. Individuals are assigned to the trajectory groups based on their posterior probabilities, and the model parameters are generated by maximum likelihood estimation (Nagin, 2014). We selected the number of trajectory groups and the trajectory shapes based on substance-specific interpretability of the trajectories and the following statistical criteria: 1) Bayesian information criterion (BIC), 2) the average of the posterior probability (APP) of group membership in a trajectory group over 0.7, 3) the odds of correct classification (OCC) based on the posterior probabilities of group membership over 5.0, and 4) a trajectory group size over 5% of the study population (Nagin & Odgers, 2010). The selection of the most optimal models is shown in the supplementary material (Table S3).

We used multinomial logistic regression analyses to estimate inequalities in the identified trajectories, looking both at bivariate and multivariate associations. All statistical analyses were performed using Stata version 16 (StataCorp LLC, College Station, TX, USA). Whilst health variables remain central to this method, the interest is in identifying different trajectories and exploring whether membership is associated with inequalities. Of key import, we do not make ‘a priori’ assumptions about whether some trajectories represent recovery and others do not – a point we return to in the discussion.

## 4 Findings

### 4.1 Approach 1: recovery as an ‘outcome’

When examining recovery as an outcome, unconditional results showed that 39-year-olds or younger (ref. group), males (OR 1.24, 95% CI 1.13–1.36), Asian/Asian British ethnic group (OR 2.30, 95% CI 1.98–2.68), never married (OR 1.73, 95% CI 1.54–1.94), those with an A-level or equivalent education (OR 1.22, 95% CI 1.01–1.49) and full-time students (OR 2.83, 95% CI 2.22–3.62) were most likely to recover from a poor physical health state (Table 2). When examined simultaneously in the same model, however, only the associations for age and gender remained similar. Ethnicity was no longer associated with recovery from poor health after adjustment of all predictors. Decreased odds of recovery from poor physical health were found for older age groups, females, widowed, divorced/separated, those with GCSE/equivalent or no educational qualification, unemployed, retired and those with family care or other employment status, after full adjustment.

**Table 2:** Odds of recovery from poor health functioning: results from multilevel logistic regression models where cross-sectional associations in Waves 1–9 were merged. Odds ratios (OR) with 95% confidence intervals (CI) are shown.

	Physical health: OR (95% CI)		Mental health: OR (95% CI)	
	Unadjusted	Fully adjusted a	Unadjusted	Fully adjusted a
**Age**				
39 years old/younger	ref.	ref.	ref.	ref.
40–64 years old	0.20* (0.17–0.22)	0.32* (0.28–0.36)	1.09* (1.03–1.16)	1.10* (1.03–1.19)
65 years old/older	0.09* (0.08–0.10)	0.25* (0.21–0.30)	1.67* (1.53–1.82)	1.66* (1.42–1.94)
**Gender**				
Female	ref.	ref.	ref.	ref.
Male	1.24* (1.13–1.36)	1.24* (1.14–1.36)	1.25* (1.18–1.33)	1.23* (1.15–1.31)
**Ethnicity**				
White	ref.	ref.	ref.	ref.
Mixed	2.20* (1.52–3.17)	0.95 (0.68–1.33)	0.80* (0.66–0.98)	0.95 (0.78–1.15)
Asian/Asian British	2.30* (1.98–2.68)	1.08 (0.93–1.25)	0.97 (0.88–1.07)	0.99 (0.90–1.10)
Black/Black British	1.76* (1.38–2.25)	1.17 (0.93–1.46)	1.08 (0.93–1.25)	1.22* (1.05–1.41)
Other ethnic group	1.34 (0.79–2.26)	0.85 (0.52–1.41)	0.77 (0.54–1.09)	0.76 (0.53–1.08)
**Marital status**				
Partnership	ref.	ref.	ref.	ref.
Widowed	0.25* (0.22–0.29)	0.63* (0.55–0.73)	1.13* (1.00–1.28)	1.06 (0.92–1.23)
Divorced/separated	0.47* (0.41–0.53)	0.68* (0.60–0.78)	0.67* (0.61–0.73)	0.77* (0.70–0.85)
Never married	1.73* (1.54–1.94)	1.11 (0.97–1.26)	0.68* (0.64–0.73)	0.78* (0.72–0.85)
**Educational attainment**				
Higher degree	ref.	ref.	ref.	ref.
A-level/equivalent	1.22* (1.01–1.49)	0.94 (0.79–1.12)	0.79* (0.71–0.87)	0.89* (0.81–0.99)
GCSE/equivalent	0.84* (0.74–0.96)	0.86* (0.76–0.96)	0.72* (0.66–0.77)	0.85* (0.79–0.92)
Other qualification	0.38* (0.29–0.49)	0.86 (0.68–1.08)	1.00 (0.77–1.21)	1.03 (0.83–1.29)
No qualification	0.28* (0.25–0.32)	0.63* (0.56–0.70)	0.75* (0.70–0.82)	0.85* (0.78–0.93)
**Employment status**				
Employed	ref.	ref.	ref.	ref.
Unemployed	0.43* (0.37–0.50)	0.41* (0.35–0.48)	0.55* (0.50–0.60)	0.59* (0.53–0.65)
Retired	0.16* (0.15–0.18)	0.33* (0.29–0.37)	1.05 (0.98–1.14)	0.75* (0.66–0.86)
Family care	0.50* (0.44–0.57)	0.47* (0.41–0.55)	0.54* (0.49–0.60)	0.60* (0.54–0.66)
Full-time student	2.83* (2.22–3.62)	1.24 (0.95–1.62)	0.70* (0.63–0.77)	0.87* (0.77–0.98)
Other	0.05* (0.05–0.06)	0.07* (0.06–0.08)	0.24* (0.22–0.27)	0.25* (0.22–0.27)

*P-value<0.05.

a Variables adjusted mutually for each other.

Concerning recovery from poor mental health state, unconditional results showed that 65-year-olds or older (OR 1.67, 95% CI 1.53–1.82), males (OR 1.25, 95% CI 1.18–1.33) and widowed (OR 1.13, 95% CI 1.00–1.28) were most likely to recover (Table 2). Similar to physical health, the results for age and gender did not change substantially when all variables were included simultaneously. Black/Black British ethnic group had the highest odds of recovery from poor mental health state (OR 1.22, 95% CI 1.05–1.41), but otherwise, ethnicity was not associated with recovery after adjustment of all predictors. Divorced/separated, never married, those with lower educational attainment (except ‘other’ qualification) and others than employed had decreased odds of recovery from poor mental health state, after full adjustment. The stepwise-adjusted results are shown in the supplementary material (Table S4).

### 4.2 Approach 2: recovery as a ‘process’

**Fig. 2:**
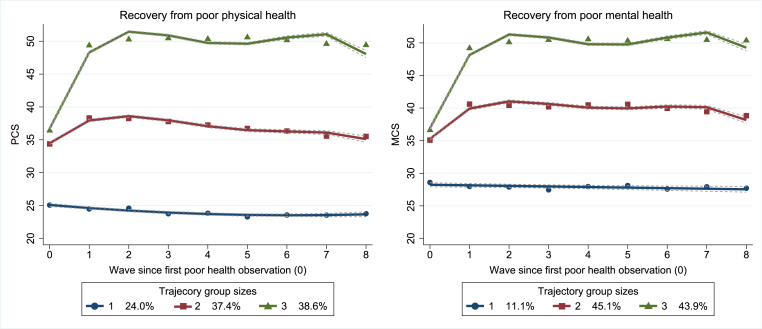
Physical and mental health trajectories since participants' first poor health observation. Trajectory groups: 1 = low-stable, 2 = moderate-stable, 3 = fast-increasing. Poor health was considered as belonging to the lowest health functioning quantile in the initial analytical sample (n = 56,118). Physical and mental health functioning were estimated using physical and mental health component summary (PCS and MCS) scores from the 12-Item Short Form Survey (SF-12). N = 18,826 for physical health and n = 22,449 for mental health.

By using GBTM, we selected 3-trajectory solutions as best-fitting models for recovery after the first poor physical and mental health observations (Fig. 2). The trajectory groups were named as ‘low-stable’ (group 1), ‘moderate-stable’ (group 2) and ‘fast-increasing’ (group 3). The patterns of the identified trajectories were somewhat parallel for physical and mental health. Approximately a quarter (24%) of the participants were assigned to the physical health trajectory group 1, which showed a consistently low health state across all waves. A similar trajectory group was also identified for mental health, comprising around one tenth (11%) of the participants. For mental health, the absolute trajectory level was somewhat higher. Roughly, equal amounts of participants were assigned to trajectory groups 2 and 3: 37% and 39% for physical health and 45% and 44% for mental health. These trajectories – especially trajectory 3 – showed substantial improvements in health between Waves 1 and 2, with achieved health status either then remaining stable or slightly worsening.

Table 3 shows the odds of belonging to trajectory groups 2 (‘moderate-stable’) and 3 (‘fast-increasing’), compared to group 1 (‘low-stable’). Concerning physical health, unconditional results showed that 39-year-olds or younger (ref. group), males (OR 1.19, 95% CI 1.10–1.28), Asian/Asian British ethnic group (OR 2.12, 95% CI 1.84–2.44), never married (OR 1.83, 95% CI 1.63–2.05) and full-time students (OR 2.46, 95% CI 1.68–3.62) had the highest odds of belonging to trajectory group 3. The patterns for the associations of predictors with trajectory group 2 were mostly similar to group 3, though the associations were more modest. When the full set of predictors were included, the associations remained similar, except for ethnicity which was no longer associated with trajectory group 3. Additionally, full-time students no longer had higher odds of belonging to trajectory groups 2 and 3, while employed had statistically significantly higher odds of belonging to these groups.

**Table 3:** Odds of belonging to physical and mental health recovery trajectory groups 2 (moderate-stable) and 3 (fast-increasing), compared to group 1 (low-stable): multinomial logistic regression models. Odds ratios (OR) with 95% confidence intervals (CI) are shown.

	Physical health: OR (95% CI)				Mental health: OR (95% CI)			
	Unadjusted		Fully adjusted a		Unadjusted		Fully adjusted a	
	Group 2 Moderate-stable	Group 3 Fast-increasing	Group 2 Moderate-stable	Group 3 Fast-increasing	Group 2 Moderate-stable	Group 3 Fast-increasing	Group 2 Moderate-stable	Group 3 Fast-increasing
**Age**								
39 years old/younger	ref.	ref.	ref.	ref.	ref.	ref.	ref.	ref.
40–64 years old	0.50* (0.44–0.57)	0.22* (0.19–0.24)	0.67* (0.57–0.79)	0.32* (0.27–0.37)	1.20* (1.09–1.31)	1.51* (1.37–1.65)	1.22* (1.07–1.38)	1.54* (1.35–1.75)
65 years old/older	0.42* (0.37–0.48)	0.09* (0.08–0.10)	0.71* (0.57–0.88)	0.23* (0.18–0.29)	2.13* (1.80–2.53)	3.63* (3.06–4.31)	1.95* (1.40–2.72)	3.23* (2.32–4.51)
**Gender**								
Female	ref.	ref.	ref.	ref.	ref.	ref.	ref.	ref.
Male	1.06 (0.98–1.14)	1.19* (1.10–1.28)	1.04 (0.95–1.14)	1.30* (1.18–1.44)	1.28* (1.17–1.41)	1.48* (1.35–1.62)	1.34* (1.20–1.49)	1.53* (1.37–1.71)
**Ethnicity**								
White	ref.	ref.	ref.	ref.	ref.	ref.	ref.	ref.
Mixed	1.12 (0.79–1.60)	2.06* (1.49–2.84)	0.94 (0.63–1.38)	1.06 (0.71–1.56)	0.69* (0.53–0.89)	0.59* (0.45–0.77)	0.86 (0.65–1.14)	0.98 (0.64–1.14)
Asian/Asian British	1.79* (1.55–2.07)	2.12* (1.84–2.44)	1.44* (1.22–1.71)	1.10 (0.92–1.31)	1.23* (1.06–1.44)	1.11 (0.96–1.30)	1.31* (1.11–1.54)	1.24* (1.05–1.47)
Black/Black British	1.08 (0.87–1.32)	1.40* (1.15–1.72)	0.90 (0.72–1.13)	0.92 (0.73–1.17)	1.39* (1.09–1.79)	1.48* (1.15–1.89)	1.69* (1.30–2.19)	1.96* (1.51–2.55)
Other ethnic group	1.87* (1.15–3.06)	1.64* (1.00–2.70)	2.02* (1.10–3.70)	1.27 (0.66–2.43)	1.19 (0.71–1.98)	0.83 (0.49–1.42)	1.33 (0.77–2.31)	0.94 (0.52–1.68)
**Marital status**								
Partnership	ref.	ref.	ref.	ref.	ref.	ref.	ref.	ref.
Widowed	0.57* (0.51–0.64)	0.21* (0.18–0.24)	0.74* (0.64–0.85)	0.56* (0.47–0.66)	1.02 (0.80–1.29)	1.31* (1.04–1.65)	0.89 (0.67–1.20)	0.98 (0.73–1.31)
Divorced/separated	0.68* (0.61–0.77)	0.46* (0.41–0.52)	0.83* (0.73–0.95)	0.68* (0.59–0.79)	0.59* (0.52–0.68)	0.48* (0.42–0.56)	0.66* (0.56–0.78)	0.59* (0.50–0.69)
Never married	1.25* (1.10–1.41)	1.83* (1.63–2.05)	1.33* (1.14–1.56)	1.34* (1.14–1.58)	0.59* (0.53–0.65)	0.44* (0.40–0.49)	0.76* (0.67–0.87)	0.66* (0.58–0.76)
**Educational attainment**								
Higher degree	ref.	ref.	ref.	ref.	ref.	ref.	ref.	ref.
A-level/equivalent	1.08 (0.88–1.31)	1.11 (0.92–1.34)	1.02 (0.83–1.26)	0.86 (0.69–1.06)	0.69* (0.58–0.81)	0.59* (0.50–0.69)	0.89 (0.75–1.07)	0.98 (0.72–1.03)
GCSE/equivalent	0.95 (0.83–1.08)	0.87* (0.77–0.99)	0.99 (0.86–1.13)	0.84* (0.73–0.96)	0.58* (0.51–0.66)	0.50* (0.44–0.57)	0.78* (0.68–0.90)	0.73* (0.64–0.84)
Other qualification	0.73* (0.58–0.91)	0.40* (0.32–0.50)	0.97 (0.77–1.22)	0.84 (0.65–1.08)	0.91 (0.62–1.34)	0.94 (0.64–1.38)	1.05 (0.70–1.56)	1.01 (0.68–1.51)
No qualification	0.61* (0.55–0.68)	0.29* (0.26–0.32)	0.83* (0.73–0.93)	0.55* (0.48–0.62)	0.63* (0.55–0.72)	0.59* (0.51–0.67)	0.81* (0.70–0.94)	0.74* (0.63–0.86)
**Employment status**								
Employed	ref.	ref.	ref.	ref.	ref.	ref.	ref.	ref.
Unemployed	0.60* (0.50–0.73)	0.36* (0.30–0.43)	0.53* (0.43–0.66)	0.30* (0.24–0.37)	0.39* (0.34–0.46)	0.27* (0.23–0.32)	0.41* (0.35–0.48)	0.30* (0.25–0.36)
Retired	0.34* (0.31–0.38)	0.10* (0.09–0.12)	0.41* (0.35–0.49)	0.24* (0.20–0.28)	0.99 (0.84–1.16)	1.21* (1.03–1.42)	0.67* (0.50–0.89)	0.61* (0.46–0.81)
Family care	0.71* (0.59–0.85)	0.47* (0.40–0.56)	0.64* (0.52–0.79)	0.43* (0.35–0.52)	0.49* (0.42–0.57)	0.32* (0.28–0.38)	0.55* (0.46–0.66)	0.41* (0.34–0.49)
Full-time student	1.27 (0.84–1.91)	2.46* (1.68–3.62)	0.68 (0.44–1.07)	0.81 (0.53–1.24)	0.44* (0.38–0.50)	0.32* (0.27–0.37)	0.55* (0.46–0.65)	0.50* (0.42–0.60)
Other	0.15* (0.13–0.17)	0.03* (0.03–0.04)	0.15* (0.13–0.17)	0.04* (0.03–0.04)	0.25* (0.22–0.28)	0.09* (0.07–0.10)	0.24* (0.20–0.28)	0.09* (0.07–0.11)

*P-value<0.05.

a Variables adjusted mutually for each other.

With respect to mental health, unconditional results showed that 65-year-olds or older (OR 1.19, 95% CI 3.06–4.31), males (OR 1.48, 95% CI 1.35–1.62), Black/Black British ethnic group (OR 1.48, 95% CI 1.15–1.89), widowed (OR 1.31, 95% CI 1.04–1.65) and retired (OR 1.21, 95% CI 1.03–1.42) had the highest odds of belonging to trajectory group 3 (Table 3). Again, similar tendencies could be seen for trajectory group 2. In addition, those with higher degree education were more likely to belong to trajectory groups 2 and 3 than those with lower educational attainment. The associations mostly remained after full adjustments. However, widowed were no longer more likely to belong to trajectory group 3, and retired had decreased odds of belonging to both trajectory groups 2 and 3 when full set of predictors were included. Employed had statistically significantly higher odds of belonging to trajectory groups 2 and 3 than other employment status groups after full adjustment. Tables S5–S6 in the supplementary material show the stepwise-adjusted results for the trajectory analyses.

## 5 Discussion

Despite a breadth of focus, large-scale research on inequalities in recovery has tended to operationalise recovery as an ‘outcome’ and something akin to ‘return to baseline’. We therefore know less about patterns and predictors when recovery is situated as something more fluid, and which varies from person to person (‘a process’). The present study is among the first to explore patterns and predictors of ‘recovery as a process’ whilst simultaneously exploring the role of methodological artefact as a possible mechanism for difference.

Analysis identified discernible trajectories whilst also highlighting the key role that demographic and socioeconomic factors play in determining those trajectories. When operationalised like this, then, recovery is seen as both heterogeneous and shaped by the socio-structural conditions of living. These conditions and determinants reflect much of what is already known. For example, it is unsurprising that marital status played a key role in differentiating mental health recovery trajectories, given that support and reciprocity with family members have been shown to be important to the recovery process (Pernice-Duca 2010). Whilst present findings for physical health may therefore seem counterintuitive, social support has produced mixed results in physical health (see Reifman, 1995, for example). Current findings for gender echo previous research which suggests that being female is associated with decreased chance of recovery (Pevalin & Goldberg, 2003). They also lend support to feminist analysis which has suggested that notions of ‘dutiful’ recovery complicate women's recovery efforts (Fullager and O'Brien 2014). Results for age and mental health appear counterintuitive in that older adults are more likely to belong to trajectories with quicker health transitions. However, it is known that first episode of mental disorder occurs before 25 in approximately 65% of cases (Solmi et al., 2021) and current findings are possibly a result of recovery intersecting with age of onset. More research would be useful here.

Current findings also raise questions concerning mechanisms. Whilst the role of employment is clear in differentiating trajectories, the literature on employment and recovery is complex. Supported employment is considered to be an evidence-based intervention in mental health (Drake & Wallach, 2020) and for some individuals, employment plays a central role in their lives and identities and confers benefit in their recovery process (Dunn et al., 2008). At the same time, there are as many employment barriers as there are employment enablers in recovery (Munro & Edward, 2008).

With respect to artefact as possible mechanism, our analysis demonstrated that the inequalities in recovery from poor health state are robust, regardless of whether recovery is operationalised as an outcome or as a process. When looked at as an outcome, being employed, especially, increased the odds of recovery both from poor physical and mental health state. Similar to Tanaka's et al. (2018) findings, employment status showed more robust associations with recovery than educational attainment. Contrary to this, individuals who were divorced/separated were less likely to recover from poor physical and mental health state. Further investigation is needed to clarify the impact of ethnicity. While people from Black, Asian and minority ethnic groups have been shown to have worse health than White British people in general (Wohland et al., 2015), only little research exists on recovery. Similar patterns for inequalities in recovery were observed when recovery was operationalised as a process and different trajectories identified, though socioeconomic gradient in recovery became more visible.

However, and notwithstanding this, a ‘soft’ version of the methodological artefact may still run true. Whilst trajectory models were primarily used to approximate recovery as a different process, they also provide a window onto what happens if outcome models are extended over a single time point. These models would suggest that recovery from poor health was more pronounced and long-lasting when the baseline state was less severe, corresponding Tanaka's et al. (2018) findings with a shorter follow-up. The models also highlight how recovery (outcome) is not a straightforward transition from poor to good health. For example, the second trajectory group showed descending curves in health after the first ‘improvement’ observation, and for physical health, the trajectory level almost returned to the same level as at the time of the first poor health observation. Taken together, this suggests that when recovery is operationalised as a simple transition from poor health state to good, we lose sight of the fact that there may exist inequalities (i) within a ‘poor health’ state, (ii) in how individuals are able to step into the path of recovery, and (iii) in how they keep on staying in a better health if they recover.

Limitations of this study need to be considered. Missing data are always a concern and potential source of bias in longitudinal research. In our analyses, missing data in predictors were rare (≤0.1% of participants), apart from education in which 16–18% of participants had missing or inapplicable data. Imputing missing educational data by creating an extra class yielded similar fully adjusted results as our main analyses (see Table S7). Concerning trajectory analyses, the trajectories were modelled with GBTM that uses maximum likelihood estimation. Thus, trajectories were identified for all participants (i.e., having health functioning data at least in 2/9 waves). We also modelled trajectories for those participants who had health functioning data in 5/9 waves, and the trajectory patterns were highly similar to our main analyses (see Fig. S1). Inequalities in recovery from poor health focused on general characteristics of study population (i.e., age, gender, ethnicity, marital status, education and employment status). However, we acknowledge that inequalities in recovery are not limited to these determinants; thus, future studies should also examine inequalities in recovery in terms of sexual identities, material resources or geography, for example. We also did not include measures of life satisfaction, which may be important indicators of recovery as a process. The SF-12 measures of physical and mental health functioning (i.e., PCS and MCS) also include some limitations. First, subjective estimates of one's health may vary depending on socioeconomic characteristics, which can affect findings on inequalities in recovery. Second, longitudinal observations in PCS and MCS may not correspond clinically measured changes in health (Simon et al., 1998). Third, the selected threshold in PCS and MCS to reflect poor health state can have small effects on the results: for instance, using quintiles instead of quartiles (another widely used threshold) may produce slightly higher estimates (e.g., Raymakers et al., 2018).

The subjective nature of the PCS and MCS measures is, however, also a strength, given that recovery itself is a personal experience including variety of meanings for individuals, and that cannot fully be captured objectively (Anthony, 1993; Meadows et al., 2020). The good reliability of the SF-12 questionnaire has been verified across study populations (Cheak-Zamora et al., 2009; Huo et al., 2018; Salyers et al., 2000). The utility in GBTM is that it explores and identifies different health trajectories without making ‘a priori’ judgments about which of them constitutes recovery. A major strength of this study is the use of a comprehensive longitudinal data on the UK population. Since our analytical samples were selected based on the available subsequent health functioning data in Waves 1–9 and focused on participants with poor health observation(s), the findings are not directly generalisable to the general UK population, however. The focus of this study was not on examining inequalities in health in general, but it is possible that health inequalities existing among the overall study population have some impact on the findings. For example, participants in the initial study sample (n = 56,118) were, on average, more often men, in partnership, had higher degree education and were more often employed than participants in the analytical samples. Thus, our results imply that (socioeconomic) inequalities in recovery further increase existing health inequalities, though the impact of ethnicity remains unclear.

## 6 Conclusions

Our research contributes to understandings of inequalities in recovery. At the same time as continuing to highlight the importance of age, gender, marital status, education and employment in recovery trajectories, this paper evidenced the need to pay close attention to the meanings of recovery. Our study also illustrates how it is possible to bridge the gap between qualitative/conceptual discussions of recovery and large-scale secondary analysis. There is a need for more population level research into the complexities of recovery – especially considering the current emphasis on ideas of recovery in the midst and after the pandemic, and concerns of widening health inequalities (i.e., Hoernke, 2020; Fauzi et al., 2020; Johnson et al., 2020; Prescott & Girard, 2020; Voinsky et al., 2020). Secondary data is well suited to address these complexities.

## Ethical statement

The Understanding Society study protocol is scrutinised by a number of research ethics committees to assure that ethical and legal obligations are respected at all times. The ethical approval statement is available from the Understanding Society websites: https://www.understandingsociety.ac.uk/documentation/mainstage/user-guides/main-survey-user-guide/ethics.

## CRediT authorship contribution statement

**Salmela Jatta:** Data curation, Formal analysis, Investigation, Methodology, Software, Visualization, Writing – original draft, Writing – review & editing. **Brunton-Smith Ian:** Methodology, Writing – original draft, Writing – review & editing. **Meadows Robert:** Conceptualization, Investigation, Methodology, Project administration, Writing – original draft, Writing – review & editing.

## Declaration of interest statement

The authors have no conflicts of interest.
