# Supplementary material for: Inequalities in recovery or methodological artefact? A comparison of models across physical and mental health functioning
Source: SSM Popul Health. 2022 Mar 5;17:101067. doi: 10.1016/j.ssmph.2022.101067 (PMC8914363; doi:10.1016/j.ssmph.2022.101067)
Supplement: Multimedia component 1 [file mmc1.docx]

**Supplementary material**

**Table S1.** Number of participants with physical and mental health functioning observations at each wave (W), and the share of participants with poor health observation and those who recovered from poor to good health. Altogether, N=56,118 in W1–9.

|  | **Period 1: W1–W2** | **Period 2: W2–W3** | **Period 3: W3–W4** | **Period 4: W4–W5** | **Period 5: W5–W6** | **Period 6: W6–W7** | **Period 7: W7–W8** | **Period 8: W8–W9** | **Person-observations, W1–W9** |
| --- | --- | --- | --- | --- | --- | --- | --- | --- | --- |
| **Health scores at both waves, n** | 27,639 | 30,307 | 33,059 | 32,540 | 29,632 | 29,691 | 30,960 | 29,009 | 242,837 |
| Poor physical health at start of period, n (%) | 5381 (19.5) | 5644 (18.6) | 6482 (19.6) | 6303 (19.4) | 5697 (19.2) | 5796 (19.5) | 6003 (19.4) | 5565 (19.2) | 46,871  (19.3) |
| *Recovered physical health at end of period, n (%)* | 1615 (30.0) | 1673 (29.6) | 2035 (31.4) | 1896 (30.1) | 1744 (30.6) | 1672 (28.9) | 1844 (30.7) | 1634 (29.4) | 14,113  (30.1) |
| Poor mental health at start of period, n (%) | 5269 (19.1) | 5854 (19.3) | 6472 (19.6) | 6336 (19.5) | 5744 (19.4) | 5785 (19.5) | 5993 (19.4) | 5566 (19.2) | 47,019  (19.4) |
| *Recovered mental health at end of period, n (%)* | 2595 (49.3) | 2877 (49.2) | 3082 (47.6) | 2916 (46.0) | 2762 (48.1) | 2787 (48.2) | 2861 (47.7) | 2532 (45.5) | 22,412  (47.7) |

**Table S2.** Odds of recovery from poor health functioning in each period (i.e., two subsequent waves) during Waves 1–9: univariate binary logistic regression models. Odds ratios are shown and statistically significant (p<0.05) results are indicated with asterisk.

|  | **Physical health (N=18,826)** | | | | | | | | **Mental health (N=22,449)** | | | | | | | |
| --- | --- | --- | --- | --- | --- | --- | --- | --- | --- | --- | --- | --- | --- | --- | --- | --- |
|  | **Period** | | | | | | | | **Period** | | | | | | | |
|  | **1** | **2** | **3** | **4** | **5** | **6** | **7** | **8** | **1** | **2** | **3** | **4** | **5** | **6** | **7** | **8** |
| **Age** |  |  |  |  |  |  |  |  |  |  |  |  |  |  |  |  |
| 39 years old/younger | ref. | ref. | ref. | ref. | ref. | ref. | ref. | ref. | ref. | ref. | ref. | ref. | ref. | ref. | ref. | ref. |
| 40-64 years old | 0.37* | 0.30* | 0.32* | 0.33* | 0.29* | 0.36* | 0.35* | 0.34* | 0.77* | 1.08 | 0.89* | 0.93 | 1.08 | 0.99 | 1.19* | 1.18* |
| 65 years old/older | 0.17* | 0.25* | 0.21* | 0.20* | 0.19* | 0.27* | 0.20* | 0.27* | 0.98 | 1.67* | 1.57* | 1.24* | 1.86* | 1.35* | 1.57* | 1.66* |
| **Gender** |  |  |  |  |  |  |  |  |  |  |  |  |  |  |  |  |
| Female | ref. | ref. | ref. | ref. | ref. | ref. | ref. | ref. | ref. | ref. | ref. | ref. | ref. | ref. | ref. | ref. |
| Male | 0.98 | 1.09 | 1.09 | 1.22 | 1.46* | 1.10 | 1.25* | 1.20* | 1.08 | 1.10 | 1.18* | 1.11* | 1.18* | 1.29* | 1.18* | 1.26* |
| **Ethnicity** |  |  |  |  |  |  |  |  |  |  |  |  |  |  |  |  |
| White | ref. | ref. | ref. | ref. | ref. | ref. | ref. | ref. | ref. | ref. | ref. | ref. | ref. | ref. | ref. | ref. |
| Mixed | 1.25 | 1.93* | 2.00* | 2.04* | 1.66* | 1.15 | 1.21 | 1.13 | 1.36 | 0.94 | 0.75 | 0.96 | 0.75 | 0.99 | 0.76 | 0.63* |
| Asian/Asian British | 1.13 | 2.12* | 1.77* | 1.87* | 2.40* | 1.76* | 2.23* | 1.64* | 0.98 | 0.99 | 1.11 | 1.04 | 0.9 | 1.00 | 1.08 | 1.10 |
| Black/Black British | 1.19 | 2.09* | 1.69* | 1.72* | 1.79* | 1.73* | 1.53* | 1.14 | 1.26 | 0.92 | 1.15 | 0.95 | 1.04 | 1.31 | 1.25 | 1.08 |
| Other ethnic group | 1.73 | 2.58* | 0.58 | 0.80 | 2.00 | 2.04* | 0.87 | 1.27 | 0.66 | 1.29 | 1.28 | 0.54 | 0.71 | 0.81 | 0.97 | 1.00 |
| **Marital status** |  |  |  |  |  |  |  |  |  |  |  |  |  |  |  |  |
| Partnership | ref. | ref. | ref. | ref. | ref. | ref. | ref. | ref. | ref. | ref. | ref. | ref. | ref. | ref. | ref. | ref. |
| Widowed | 0.43* | 0.46* | 0.43* | 0.46* | 0.46* | 0.52* | 0.41* | 0.58* | 0.93 | 1.31* | 1.18 | 0.95 | 1.33* | 0.98 | 1.05 | 1.09 |
| Divorced/  separated | 0.67* | 0.63* | 0.62* | 0.60* | 0.68* | 0.52* | 0.48* | 0.59* | 0.68* | 0.72* | 0.70* | 0.62* | 0.74* | 0.68* | 0.66* | 0.74* |
| Never married | 1.31* | 1.50* | 1.49* | 1.54* | 1.50* | 1.26* | 1.28* | 1.20* | 0.90 | 0.70* | 0.82* | 0.77* | 0.74* | 0.81* | 0.68* | 0.66* |
| **Educational attainment** |  |  |  |  |  |  |  |  |  |  |  |  |  |  |  |  |
| Higher degree | ref. | ref. | ref. | ref. | ref. | ref. | ref. | ref. | ref. | ref. | ref. | ref. | ref. | ref. | ref. | ref. |
| A-level/equivalent | 1.01 | 1.21 | 1.22 | 1.12 | 1.23 | 1.22 | 1.24 | 1.11 | 0.98 | 0.74* | 0.87 | 0.79* | 0.80* | 0.88 | 0.93 | 0.71* |
| GCSE/equivalent | 0.96 | 0.95 | 0.98 | 0.83* | 0.95 | 0.88 | 0.90 | 0.82* | 0.85* | 0.74* | 0.81* | 0.84* | 0.77* | 0.76* | 0.78* | 0.67* |
| Other qualification | 0.47* | 0.86 | 0.62* | 0.60* | 0.88 | 0.54* | 0.53* | 0.76 | 0.73 | 1.08 | 0.83 | 1.27 | 0.95 | 1.06 | 1.07 | 1.38 |
| No qualification | 0.41* | 0.61* | 0.57* | 0.53* | 0.63* | 0.56* | 0.54* | 0.64* | 0.74* | 0.80* | 0.72* | 0.80* | 0.88 | 0.85* | 0.92 | 0.84* |
| **Employment status** |  |  |  |  |  |  |  |  |  |  |  |  |  |  |  |  |
| Employed | ref. | ref. | ref. | ref. | ref. | ref. | ref. | ref. | ref. | ref. | ref. | ref. | ref. | ref. | ref. | ref. |
| Unemployed | 0.63* | 0.83 | 0.76* | 0.60* | 0.60* | 0.55* | 0.55* | 0.49* | 0.62* | 0.59* | 0.62* | 0.60* | 0.65* | 0.56* | 0.62* | 0.57* |
| Retired | 0.19* | 0.35* | 0.29* | 0.26* | 0.30* | 0.36* | 0.28* | 0.35* | 0.86 | 1.08 | 1.03 | 0.95 | 1.29* | 0.96 | 1.04 | 1.16* |
| Family care | 0.55* | 0.87 | 0.66* | 0.68* | 0.62* | 0.77* | 0.71* | 0.75* | 0.66* | 0.62* | 0.54* | 0.66* | 0.57* | 0.54* | 0.57* | 0.66* |
| Full-time student | 1.48 | 3.15* | 2.65* | 2.74* | 2.60* | 1.83* | 4.12* | 2.33* | 1.17 | 0.79* | 0.94 | 0.77* | 0.78* | 0.95 | 0.71* | 0.55* |
| Other | 0.10* | 0.14* | 0.09* | 0.09* | 0.11* | 0.12* | 0.12* | 0.11* | 0.26* | 0.30* | 0.28* | 0.33* | 0.30* | 0.30* | 0.27* | 0.30* |

**Table S3.** Model selection stages for the most optimal (underlined) physical and mental health recovery trajectory groups.

| **Physical health (N=18,826)** | | | | | | **Mental health (N=22,449)** | | | | | |
| --- | --- | --- | --- | --- | --- | --- | --- | --- | --- | --- | --- |
| **Number of groups** | **Trajectory shapes ^a^** | **BIC** | **Group 1 (%)** | **Group 2 (%)** | **Group 3 (%)** | **Number of groups** | **Trajectory shapes ^a^** | **BIC** | **Group 1 (%)** | **Group 2 (%)** | **Group 3 (%)** |
| 2 | 4 4 | -347,435 | 40.3 | 59.7 |  | 2 | 4 4 | -413,120 | 31.5 | 68.5 |  |
| 3 | 4 4 4 | -340,782 | 24.1 | 37.4 | 38.4 | 3 | 4 4 4 | -409,813 | 11.8 | 44.5 | 43.7 |
| 4 | 4 4 4 4 | Model is not interpretable | | | | 4 | 4 4 4 4 | Model is not interpretable | | | |
| 3 | 3 3 3 | -341,466 | 24.0 | 37.0 | 38.9 | 3 | 3 3 3 | -410,640 | 11.6 | 43.9 | 44.5 |
| 3 | 2 2 2 | -343,178 | 23.7 | 36.27 | 40.0 | 3 | 2 2 2 | -412,554 | 11.3 | 43.6 | 45.1 |
| 3 | 3 4 4 | -340,777 | 24.1 | 37.4 | 38.4 | 3 | 3 4 4 | -409,809 | 11.8 | 44.5 | 43.7 |
| 3 ^b^ | 2 4 4 | -340,773 | 24.1 | 37.5 | 38.4 | 3 | 2 4 4 | -409,807 | 11.9 | 44.5 | 43.7 |
| 3 | 1 4 4 | -340,778 | 24.1 | 37.5 | 38.4 | 3 ^c^ | 1 4 4 | -409,806 | 11.9 | 44.5 | 43.6 |
| 3 | 2 3 4 | -340,826 | 23.9 | 37.1 | 39.0 | 3 | 1 3 4 | -409,934 | 11.4 | 42.9 | 45.7 |
| 3 | 2 4 3 | -341,379 | 24.3 | 37.5 | 38.2 | 3 | 1 4 3 | -410,389 | 12.7 | 46.2 | 41.1 |

^a^ Trajectory shapes: 0=intercept, 1=linear, 2=quadratic, 3=cubic, 4=quartic.

^b^ The APPs of group membership in trajectory groups 1, 2 and 3 are 0.93, 0.86 and 0.91, respectively. The OCCs are 42.2, 10.3 and 15.6, respectively.

^c^ The APPs of group membership in trajectory groups 1, 2, and 3 are 0.87, 0.81 and 0.85, respectively. The OCCs are 52.2, 5.1 and 7.1, respectively.

**Table S4.** Odds of recovery from poor health functioning between two subsequent waves: multilevel logistic regression models. Odds ratios (OR) with 95% confidence intervals (CI) are shown.

|  | **Physical health: OR (95% CI)** | | | | **Mental health: OR (95% CI)** | | | |
| --- | --- | --- | --- | --- | --- | --- | --- | --- |
|  | **Unadjusted** | **Adjusted for age and gender** | **Adjusted for age, gender, ethnicity and marital status** | **Fully adjusted ^a^** | **Unadjusted** | **Adjusted for age and gender** | **Adjusted for age, gender, ethnicity and marital status** | **Fully adjusted ^a^** |
|  |  |  |  |  |  |  |  |  |
| **Age** |  |  |  |  |  |  |  |  |
| 39 years old/younger | ref. | ref. | ref. | ref. | ref. | ref. | ref. | ref. |
| 40-64 years old | 0.20* (0.17-0.22) | 0.19* (0.17-0.21) | 0.21* (0.19-0.24) | 0.32* (0.28-0.36) | 1.09* (1.03-1.16) | 1.09* (1.02-1.15) | 1.02 (0.95-1.09) | 1.10* (1.03-1.19) |
| 65 years old/older | 0.09* (0.08-0.10) | 0.09* (0.08-0.10) | 0.11* (0.10-0.13) | 0.25* (0.21-0.30) | 1.67* (1.53-1.82) | 1.66* (1.52-1.81) | 1.51* (1.37-1.67) | 1.66* (1.42-1.94) |
| **Gender** |  |  |  |  |  |  |  |  |
| Female | ref. | ref. | ref. | ref. | ref. | ref. | ref. | ref. |
| Male | 1.24* (1.13-1.36) | 1.39* (1.28-1.52) | 1.26* (1.16-1.38) | 1.24* (1.14-1.36) | 1.25* (1.18-1.33) | 1.24* (1.17-1.32) | 1.23* (1.16-1.31) | 1.23* (1.15-1.31) |
| **Ethnicity** |  |  |  |  |  |  |  |  |
| White | ref. | ref. | ref. | ref. | ref. | ref. | ref. | ref. |
| Mixed | 2.20* (1.52-3.17) | 1.20 (0.85-1.69) | 1.25 (0.88-1.76) | 0.95 (0.68-1.33) | 0.80* (0.66-0.98) | 0.87 (0.71-1.06) | 0.95 (0.78-1.16) | 0.95 (0.78-1.15) |
| Asian/Asian British | 2.30* (1.98-2.68) | 1.27* (1.09-1.46) | 1.22* (1.05-1.41) | 1.08 (0.93-1.25) | 0.97 (0.88-1.07) | 1.02 (0.93-1.13) | 1.00 (0.91-1.10) | 0.99 (0.90-1.10) |
| Black/Black British | 1.76* (1.38-2.25) | 1.30* (1.03-1.64) | 1.38* (1.10-1.75) | 1.17 (0.93-1.46) | 1.08 (0.93-1.25) | 1.14 (0.98-1.33) | 1.24* (1.07-1.44) | 1.22* (1.05-1.41) |
| Other ethnic group | 1.34 (0.79-2.26) | 0.92 (0.56-1.51) | 0.91 (0.56-1.50) | 0.85 (0.52-1.41) | 0.77 (0.54-1.09) | 0.80 (0.56-1.13) | 0.82 (0.58-1.16) | 0.76 (0.53-1.08) |
| **Marital status** |  |  |  |  |  |  |  |  |
| Partnership | ref. | ref. | ref. | ref. | ref. | ref. | ref. | ref. |
| Widowed | 0.25* (0.22-0.29) | 0.48* (0.42-0.54) | 0.48* (0.42-0.54) | 0.63* (0.55-0.73) | 1.13* (1.00-1.28) | 0.94 (0.82-1.08) | 0.94 (0.82-1.07) | 1.06 (0.92-1.23) |
| Divorced/  separated | 0.47* (0.41-0.53) | 0.55* (0.48-0.62) | 0.54* (0.48-0.62) | 0.68* (0.60-0.78) | 0.67* (0.61-0.73) | 0.68* (0.62-0.74) | 0.68* (0.62-0.74) | 0.77* (0.70-0.85) |
| Never married | 1.73* (1.54-1.94) | 0.90* (0.80-1.02) | 0.89 (0.79-1.01) | 1.11 (0.97-1.26) | 0.68* (0.64-0.73) | 0.72* (0.67-0.77) | 0.71* (0.66-0.76) | 0.78* (0.72-0.85) |
| **Educational attainment** |  |  |  |  |  |  |  |  |
| Higher degree | ref. | ref. | ref. | ref. | ref. | ref. | ref. | ref. |
| A-level/  equivalent | 1.22* (1.01-1.49) | 0.90 (0.75-1.09) | 0.91 (0.76-1.10) | 0.94 (0.79-1.12) | 0.79* (0.71-0.87) | 0.81* (0.73-0.90) | 0.86* (0.77-0.95) | 0.89* (0.81-0.99) |
| GCSE/equivalent | 0.84* (0.74-0.96) | 0.74* (0.65-0.84) | 0.75* (0.66-0.85) | 0.86* (0.76-0.96) | 0.72* (0.66-0.77) | 0.72* (0.67-0.78) | 0.74* (0.69-0.81) | 0.85* (0.79-0.92) |
| Other qualification | 0.38* (0.29-0.49) | 0.59* (0.46-0.76) | 0.63* (0.49-0.81) | 0.86 (0.68-1.08) | 1.00 (0.77-1.21) | 0.81 (0.64-1.02) | 0.81 (0.65-1.02) | 1.03 (0.83-1.29) |
| No qualification | 0.28* (0.25-0.32) | 0.42* (0.37-0.47) | 0.44* (0.39-0.50) | 0.63* (0.56-0.70) | 0.75* (0.70-0.82) | 0.65* (0.60-0.71) | 0.67* (0.61-0.73) | 0.85* (0.78-0.93) |
| **Employment status** |  |  |  |  |  |  |  |  |
| Employed | ref. | ref. | ref. | ref. | ref. | ref. | ref. | ref. |
| Unemployed | 0.43* (0.37-0.50) | 0.38* (0.33-0.44) | 0.39* (0.33-0.45) | 0.41* (0.35-0.48) | 0.55* (0.50-0.60) | 0.55* (0.50-0.60) | 0.58* (0.52-0.63) | 0.59* (0.53-0.65) |
| Retired | 0.16* (0.15-0.18) | 0.29* (0.26-0.33) | 0.30* (0.27-0.34) | 0.33* (0.29-0.37) | 1.05 (0.98-1.14) | 0.74* (0.66-0.84) | 0.74* (0.65-0.83) | 0.75* (0.66-0.86) |
| Family care | 0.50* (0.44-0.57) | 0.46* (0.40-0.53) | 0.45* (0.39-0.52) | 0.47* (0.41-0.55) | 0.54* (0.49-0.60) | 0.58* (0.53-0.64) | 0.56* (0.51-0.62) | 0.60* (0.54-0.66) |
| Full-time student | 2.83* (2.22-3.62) | 1.31* (1.02-1.69) | 1.25 (0.96-1.62) | 1.24 (0.95-1.62) | 0.70* (0.63-0.77) | 0.76* (0.69-0.85) | 0.85* (0.76-0.95) | 0.87* (0.77-0.98) |
| Other | 0.05* (0.05-0.06) | 0.06* (0.05-0.07) | 0.06* (0.05-0.07) | 0.07* (0.06-0.08) | 0.24* (0.22-0.27) | 0.23* (0.21-0.26) | 0.24* (0.22-0.27) | 0.25* (0.22-0.27) |

^a^ Variables mutually adjusted for each other.

*P-value<0.05.

**Table S5**. Odds of belonging to physical health recovery trajectory groups 2 (moderate-stable) and 3 (fast-increasing), compared to group 1 (low-stable): multinomial logistic regression models. Odds ratios (OR) with 95% confidence intervals (CI) are shown.

|  | **Physical health: OR (95% CI)** | | | | | | | |
| --- | --- | --- | --- | --- | --- | --- | --- | --- |
|  | **Unadjusted** | | **Adjusted for age and gender** | | **Adjusted for age, gender, ethnicity and marital status** | | **Fully adjusted ^a^** | |
|  | **Group 2**  **Moderate-stable** | **Group 3**  **Fast-increasing** | **Group 2**  **Moderate-stable** | **Group 3**  **Fast-increasing** | **Group 2**  **Moderate-stable** | **Group 3**  **Fast-increasing** | **Group 2**  **Moderate-stable** | **Group 3**  **Fast-increasing** |
| **Age** |  |  |  |  |  |  |  |  |
| 39 years old/younger | ref. | ref. | ref. | ref. | ref. | ref. | ref. | ref. |
| 40-64 years old | 0.50*  (0.44-0.57) | 0.22*  (0.19-0.24) | 0.50*  (0.44-0.57) | 0.21*  (0.19-0.24) | 0.56*  (0.49-0.64) | 0.24*  (0.21-0.27) | 0.67*  (0.57-0.79) | 0.32*  (0.27-0.37) |
| 65 years old/older | 0.42*  (0.37-0.48) | 0.09*  (0.08-0.10) | 0.41*  (0.36-0.47) | 0.09*  (0.08-0.10) | 0.52*  (0.45-0.60) | 0.12*  (0.10-0.13) | 0.71*  (0.57-0.88) | 0.23*  (0.18-0.29) |
| **Gender** |  |  |  |  |  |  |  |  |
| Female | ref. | ref. | ref. | ref. | ref. | ref. | ref. | ref. |
| Male | 1.06  (0.98-1.14) | 1.19*  (1.10-1.28) | 1.10*  (1.02-1.19) | 1.37*  (1.26-1.48) | 1.01  (0.93-1.09) | 1.22*  (1.12-1.32) | 1.04  (0.95-1.14) | 1.30*  (1.18-1.44) |
| **Ethnicity** |  |  |  |  |  |  |  |  |
| White | ref. | ref. | ref. | ref. | ref. | ref. | ref. | ref. |
| Mixed | 1.12  (0.79-1.60) | 2.06*  (1.49-2.84) | 0.96  (0.67-1.37) | 1.22  (0.87-1.71) | 0.97  (0.68-1.39) | 1.28  (0.91-1.81) | 0.94  (0.63-1.38) | 1.06  (0.71-1.56) |
| Asian/Asian British | 1.79*  (1.55-2.07) | 2.12*  (1.84-2.44) | 1.53*  (1.32-1.78) | 1.26*  (1.08-1.46) | 1.51*  (1.30-1.75) | 1.22*  (1.05-1.42) | 1.44*  (1.22-1.71) | 1.10  (0.92-1.31) |
| Black/Black British | 1.08  (0.87-1.32) | 1.40*  (1.15-1.72) | 1.01  (0.81-1.24) | 1.10  (0.89-1.36) | 1.02  (0.83-1.27) | 1.15  (0.93-1.43) | 0.90  (0.72-1.13) | 0.92  (0.73-1.17) |
| Other ethnic group | 1.87*  (1.15-3.06) | 1.64*  (1.00-2.70) | 1.70*  (1.04-2.78) | 1.16  (0.69-1.94) | 1.70*  (1.04-2.78) | 1.16  (0.69-1.95) | 2.02*  (1.10-3.70) | 1.27  (0.66-2.43) |
| **Marital status** |  |  |  |  |  |  |  |  |
| Partnership | ref. | ref. | ref. | ref. | ref. | ref. | ref. | ref. |
| Widowed | 0.57*  (0.51-0.64) | 0.21*  (0.18-0.24) | 0.64*  (0.57-0.72) | 0.41*  (0.35-0.47) | 0.64*  (0.57-0.72) | 0.41*  (0.35-0.47) | 0.74*  (0.64-0.85) | 0.56*  (0.47-0.66) |
| Divorced/  separated | 0.68*  (0.61-0.77) | 0.46*  (0.41-0.52) | 0.70*  (0.63-0.79) | 0.51*  (0.45-0.58) | 0.71*  (0.64-0.80) | 0.51*  (0.45-0.58) | 0.83*  (0.73-0.95) | 0.68*  (0.59-0.79) |
| Never married | 1.25*  (1.10-1.41) | 1.83*  (1.63-2.05) | 1.01  (0.89-1.14) | 0.95  (0.84-1.08) | 1.03  (0.91-1.18) | 0.94  (0.83-1.07) | 1.33*  (1.14-1.56) | 1.34*  (1.14-1.58) |
| **Educational attainment** |  |  |  |  |  |  |  |  |
| Higher degree | ref. | ref. | ref. | ref. | ref. | ref. | ref. | ref. |
| A-level/  equivalent | 1.08  (0.88-1.31) | 1.11  (0.92-1.34) | 0.97  (0.80-1.19) | 0.82*  (0.67-1.00) | 0.98  (0.80-1.20) | 0.83*  (0.68-1.01) | 1.02  (0.83-1.26) | 0.86  (0.69-1.06) |
| GCSE/equivalent | 0.95  (0.83-1.08) | 0.87*  (0.77-0.99) | 0.91  (0.79-1.03) | 0.75*  (0.66-0.85) | 0.92  (0.81-1.05) | 0.75*  (0.66-0.85) | 0.99  (0.86-1.13) | 0.84*  (0.73-0.96) |
| Other qualification | 0.73*  (0.58-0.91) | 0.40*  (0.32-0.50) | 0.80*  (0.64-1.00) | 0.59*  (0.46-0.75) | 0.81  (0.65-1.02) | 0.62*  (0.49-0.79) | 0.97  (0.77-1.22) | 0.84  (0.65-1.08) |
| No qualification | 0.61*  (0.55-0.68) | 0.29*  (0.26-0.32) | 0.66*  (0.59-0.74) | 0.38*  (0.34-0.43) | 0.68*  (0.61-0.76) | 0.40*  (0.35-0.45) | 0.83*  (0.73-0.93) | 0.55*  (0.48-0.62) |
| **Employment status** |  |  |  |  |  |  |  |  |
| Employed | ref. | ref. | ref. | ref. | ref. | ref. | ref. | ref. |
| Unemployed | 0.60*  (0.50-0.73) | 0.36*  (0.30-0.43) | 0.55*  (0.45-0.66) | 0.27*  (0.22-0.33) | 0.52*  (0.43-0.64) | 0.27*  (0.19-0.26) | 0.53*  (0.43-0.66) | 0.30*  (0.24-0.37) |
| Retired | 0.34*  (0.31-0.38) | 0.10*  (0.09-0.12) | 0.40*  (0.34-0.46) | 0.22*  (0.19-0.25) | 0.41*  (0.35-0.47) | 0.40*  (0.33-0.48) | 0.41*  (0.35-0.49) | 0.24*  (0.20-0.28) |
| Family care | 0.71*  (0.59-0.85) | 0.47*  (0.40-0.56) | 0.68*  (0.57-0.82) | 0.41*  (0.34-0.49) | 0.64*  (0.53-0.77) | 0.40*  (0.33-0.48) | 0.64*  (0.52-0.79) | 0.43*  (0.35-0.52) |
| Full-time student | 1.27  (0.84-1.91) | 2.46*  (1.68-3.62) | 0.85  (0.56-1.30) | 0.95  (0.64-1.42) | 0.71  (0.46-1.10) | 0.81  (0.54-1.22) | 0.68  (0.44-1.07) | 0.81  (0.53-1.24) |
| Other | 0.15*  (0.13-0.17) | 0.03*  (0.03-0.04) | 0.15*  (0.13-0.17) | 0.03*  (0.03-0.04) | 0.14*  (0.13-0.17) | 0.03*  (0.03-0.04) | 0.15*  (0.13-0.17) | 0.04*  (0.03-0.04) |

^a^ Variables mutually adjusted for each other.

*P-value<0.05.

**Table S6**. Odds of belonging to mental health recovery trajectory groups 2 (moderate-stable) and 3 (fast-increasing), compared to group 1 (low-stable): multinomial logistic regression models. Odds ratios (OR) with 95% confidence intervals (CI) are shown.

|  | **Mental health: OR (95% CI)** | | | | | | | |
| --- | --- | --- | --- | --- | --- | --- | --- | --- |
|  | **Unadjusted** | | **Adjusted for age and gender** | | **Adjusted for age, gender, ethnicity and marital status** | | **Fully adjusted ^a^** | |
|  | **Group 2**  **Moderate-stable** | **Group 3**  **Fast-increasing** | **Group 2**  **Moderate-stable** | **Group 3**  **Fast-increasing** | **Group 2**  **Moderate-stable** | **Group 3**  **Fast-increasing** | **Group 2**  **Moderate-stable** | **Group 3**  **Fast-increasing** |
| **Age** |  |  |  |  |  |  |  |  |
| 39 years old/younger | ref. | ref. | ref. | ref. | ref. | ref. | ref. | ref. |
| 40-64 years old | 1.20*  (1.09-1.31) | 1.51*  (1.37-1.65) | 1.19*  (1.09-1.31) | 1.50*  1.36-1.64) | 1.10  (0.99-1.23) | 1.33*  1.19-1.48) | 1.22*  (1.07-1.38) | 1.54*  (1.35-1.75) |
| 65 years old/older | 2.13*  (1.80-2.53) | 3.63*  (3.06-4.31) | 2.12*  (1.78-2.52) | 3.59*  (3.03-4.26) | 2.04*  (1.67-2.48) | 3.22*  2.64-3.91) | 1.95*  (1.40-2.72) | 3.23*  (2.32-4.51) |
| **Gender** |  |  |  |  |  |  |  |  |
| Female | ref. | ref. | ref. | ref. | ref. | ref. | ref. | ref. |
| Male | 1.28*  (1.17-1.41) | 1.48*  (1.35-1.62) | 1.27*  (1.16-1.40) | 1.45*  (1.32-1.60) | 1.25*  (1.14-1.37) | 1.43*  (1.30-1.57) | 1.34*  (1.20-1.49) | 1.53*  (1.37-1.71) |
| **Ethnicity** |  |  |  |  |  |  |  |  |
| White | ref. | ref. | ref. | ref. | ref. | ref. | ref. | ref. |
| Mixed | 0.69*  (0.53-0.89) | 0.59*  (0.45-0.77) | 0.76*  (0.59-0.99) | 0.72*  (0.55-0.94) | 0.84  (0.65-1.09) | 0.83  (0.63-1.08) | 0.86  (0.65-1.14) | 0.98  (0.64-1.14) |
| Asian/Asian British | 1.23*  (1.06-1.44) | 1.11  (0.96-1.30) | 1.32*  (1.13-1.54) | 1.27*  (1.09-1.49) | 1.29*  (1.11-1.50) | 1.23*  (1.05-1.44) | 1.31*  (1.11-1.54) | 1.24*  (1.05-1.47) |
| Black/Black British | 1.39*  (1.09-1.79) | 1.48*  (1.15-1.89) | 1.49*  (1.16-1.91) | 1.68*  (1.31-2.15) | 1.66*  (1.29-2.13) | 1.96*  (1.52-2.52) | 1.69*  (1.30-2.19) | 1.96*  (1.51-2.55) |
| Other ethnic group | 1.19  (0.71-1.98) | 0.83  (0.49-1.42) | 1.25  (0.75-2.08) | 0.93  (0.54-1.58) | 1.26  (0.76-2.11) | 0.95  (0.56-1.62) | 1.33  (0.77-2.31) | 0.94  (0.52-1.68) |
| **Marital status** |  |  |  |  |  |  |  |  |
| Partnership | ref. | ref. | ref. | ref. | ref. | ref. | ref. | ref. |
| Widowed | 1.02  (0.80-1.29) | 1.31*  (1.04-1.65) | 0.74*  (0.57-0.95) | 0.77*  (0.60-1.0) | 0.73*  (0.570.95) | 0.77*  (0.60-1.00) | 0.89  (0.67-1.20) | 0.98  (0.73-1.31) |
| Divorced/separated | 0.59*  (0.52-0.68) | 0.48*  (0.42-0.56) | 0.59*  (0.51-0.68) | 0.46*  (0.40-0.54) | 0.59*  (0.51-0.68) | 0.46*  (0.40-0.53) | 0.66*  (0.56-0.78) | 0.59*  (0.50-0.69) |
| Never married | 0.59*  (0.53-0.65) | 0.44*  (0.40-0.49) | 0.64*  (0.57-0.71) | 0.54*  (0.49-0.60) | 0.63*  (0.57-0.71) | 0.53*  (0.48-0.59) | 0.76*  (0.67-0.87) | 0.66*  (0.58-0.76) |
| **Educational attainment** |  |  |  |  |  |  |  |  |
| Higher degree | ref. | ref. | ref. | ref. | ref. | ref. | ref. | ref. |
| A-level/equivalent | 0.69*  (0.58-0.81) | 0.59*  (0.50-0.69) | 0.72*  (0.61-0.85) | 0.65*  (0.55-0.77) | 0.79*  (0.66-0.94) | 0.74*  (0.62-0.88) | 0.89  (0.75-1.07) | 0.98  (0.72-1.03) |
| GCSE/equivalent | 0.58*  (0.51-0.66) | 0.50*  (0.44-0.57) | 0.59*  (0.52-0.67) | 0.51*  (0.45-0.58) | 0.64*  (0.56-0.73) | 0.57*  (0.50-0.65) | 0.78*  (0.68-0.90) | 0.73*  (0.64-0.84) |
| Other qualification | 0.91  (0.62-1.34) | 0.94  (0.64-1.38) | 0.76  (0.52-1.13) | 0.66*  (0.44-1.00) | 0.76  (0.51-1.12) | 0.65*  (0.44-0.96) | 1.05  (0.70-1.56) | 1.01  (0.68-1.51) |
| No qualification | 0.63*  (0.55-0.72) | 0.59*  (0.51-0.67) | 0.53*  (0.46-0.61) | 0.42*  (0.37-0.49) | 0.57*  (0.49-0.65) | 0.45*  (0.39-0.52) | 0.81*  (0.70-0.94) | 0.74*  (0.63-0.86) |
| **Employment status** |  |  |  |  |  |  |  |  |
| Employed | ref. | ref. | ref. | ref. | ref. | ref. | ref. | ref. |
| Unemployed | 0.39*  (0.34-0.46) | 0.27*  (0.23-0.32) | 0.40*  (0.34-0.46) | 0.28*  (0.24-0.33) | 0.42*  (0.36-0.48) | 0.30*  (0.26-0.35) | 0.41*  (0.35-0.48) | 0.30*  (0.25-0.36) |
| Retired | 0.99  (0.84-1.16) | 1.21*  (1.03-1.42) | 0.60*  (0.47-0.76) | 0.54*  (0.43-0.70) | 0.61*  (0.48-0.78) | 0.55*  (0.43-0.70) | 0.67*  (0.50-0.89) | 0.61*  (0.46-0.81) |
| Family care | 0.49*  (0.42-0.57) | 0.32*  (0.28-0.38) | 0.56*  (0.47-0.65) | 0.40*  (0.34-0.47) | 0.51*  (0.44-0.60) | 0.36*  (0.31-0.43) | 0.55*  (0.46-0.66) | 0.41*  (0.34-0.49) |
| Full-time student | 0.44*  (0.38-0.50) | 0.32*  (0.27-0.37) | 0.50*  (0.43-0.59) | 0.42*  (0.36-0.49) | 0.57*  (0.48-0.67) | 0.51*  (0.43-0.60) | 0.55*  (0.46-0.65) | 0.50*  (0.42-0.60) |
| Other | 0.25*  (0.22-0.28) | 0.09*  (0.07-0.10) | 0.22*  (0.20-0.26) | 0.07*  (0.06-0.09) | 0.24*  (0.21-0.27) | 0.08*  (0.07-0.09) | 0.24*  (0.20-0.28) | 0.09*  (0.07-0.11) |

^a^ Variables mutually adjusted for each other.

*P-value<0.05.

**Table S7.** Odds of recovery from poor health functioning (‘recovery as an outcome’) and odds of belonging to health functioning trajectory groups 2 (moderate-stable) and 3 (fast-increasing), compared to group 1 (low-stable) (‘recovery as a process’): fully adjusted ^a^ results, where missing values in education have been imputed. Odds ratios (OR) with 95% confidence intervals (CI) are shown.

|  | **Recovery as an outcome**  **(multilevel logistic regression models)** | | **Recovery as a process**  **(multinomial logistic regression models)** | | | |
| --- | --- | --- | --- | --- | --- | --- |
|  | **Physical health:**  **OR (95% CI)** | **Mental health:**  **OR (95% CI)** | **Physical health: OR (95% CI)** | | **Mental health: OR (95% CI)** | |
|  |  |  | **Group 2**  **Moderate-stable** | **Group 3**  **Fast-increasing** | **Group 2**  **Moderate-stable** | **Group 3**  **Fast-increasing** |
| **Age** |  |  |  |  |  |  |
| 39 years old/younger | ref. | ref. | ref. | ref. | ref. | ref. |
| 40-64 years old | 0.32* (0.28-0.36) | 1.14* (1.07-1.22) | 0.68* (0.58-0.79) | 0.32* (0.27-0.37) | 1.28* (1.14-1.44) | 1.65* (1.47-1.86) |
| 65 years old/older | 0.25* (0.21-0.29) | 1.65* (1.43-1.90) | 0.69* (0.57-0.84) | 0.22* (0.18-0.27) | 2.19* (1.63-2.93) | 3.51* (2.62-4.71) |
| **Gender** |  |  |  |  |  |  |
| Female | ref. | ref. | ref. | ref. | ref. | ref. |
| Male | 1.28* (1.18-1.38) | 1.22* (1.15-1.29) | 1.10* (1.01-1.19) | 1.34* (1.22-1.47) | 1.31* (1.19-1.45) | 1.50* (1.36-1.67) |
| **Ethnicity** |  |  |  |  |  |  |
| White | ref. | ref. | ref. | ref. | ref. | ref. |
| Mixed | 1.03 (0.74-1.43) | 0.95 (0.79-1.16) | 0.90 (0.62-1.31) | 1.11 (0.76-1.61) | 0.86 (0.66-1.13) | 0.84 (0.64-1.12) |
| Asian/Asian British | 1.10 (0.96-1.27) | 1.01 (0.92-1.12) | 1.41* (1.21-1.65) | 1.12 (0.95-1.32) | 1.32* (1.13-1.54) | 1.27* (1.08-1.49) |
| Black/Black British | 1.15 (0.93-1.43) | 1.22* (1.05-1.42) | 0.88 (0.70-1.10) | 0.90 (0.72-1.14) | 1.67* (1.30-2.16) | 1.96* (1.51-2.54) |
| Other ethnic group | 0.83 (0.52-1.33) | 0.84 (0.60-1.17) | 1.63 (0.98-2.72) | 1.04 (0.59-1.82) | 1.30 (0.78-2.19) | 1.02 (0.59-1.76) |
| **Marital status** |  |  |  |  |  |  |
| Partnership | ref. | ref. | ref. | ref. | ref. | ref. |
| Widowed | 0.61* (0.53-0.69) | 1.04 (0.91-1.18) | 0.74* (0.66-0.84) | 0.53* (0.45-0.61) | 0.88 (0.68-1.14) | 0.98 (0.75-1.27) |
| Divorced/separated | 0.67* (0.59-0.75) | 0.75* (0.69-0.82) | 0.84* (0.74-0.95) | 0.65* (0.57-0.74) | 0.69* (0.59-0.80) | 0.57* (0.49-0.66) |
| Never married | 1.06 (0.94-1.19) | 0.78* (0.72-0.84) | 1.32* (1.15-1.52) | 1.28* (1.11-1.49) | 0.76* (0.67-0.86) | 0.66* (0.59-0.75) |
| **Educational attainment** |  |  |  |  |  |  |
| Higher degree | ref. | ref. | ref. | ref. | ref. | ref. |
| A-level/equivalent | 0.94 (0.79-1.12) | 0.89* (0.81-0.99) | 1.01 (0.82-1.25) | 0.85 (0.69-1.06) | 0.88 (0.74-1.05) | 0.85 (0.71-1.01) |
| GCSE/equivalent | 0.85* (0.76-0.96) | 0.85* (0.79-0.92) | 0.98 (0.85-1.12) | 0.83* (0.72-0.96) | 0.77* (0.67-0.88) | 0.73* (0.63-0.83) |
| Other qualification | 0.88 (0.70-1.10) | 1.04 (0.84-1.30) | 0.97 (0.77-1.22) | 0.85 (0.66-1.09) | 1.03 (0.69-1.53) | 1.02 (0.68-1.52) |
| No qualification | 0.64* (0.57-0.72) | 0.87* (0.80-0.94) | 0.82* (0.73-0.93) | 0.55* (0.48-0.62) | 0.79* (0.69-0.92) | 0.73* (0.63-0.85) |
| Missing value | 0.73* (0.64-0.82) | 0.91* (0.83-1.00) | 0.90 (0.79-1.03) | 0.65* (0.56-0.75) | 0.91 (0.78-1.07) | 0.76* (0.65-0.89) |
| **Employment status** |  |  |  |  |  |  |
| Employed | ref. | ref. | ref. | ref. | ref. | ref. |
| Unemployed | 0.41* (0.35-0.47) | 0.59* (0.54-0.65) | 0.54* (0.44-0.66) | 0.29* (0.24-0.36) | 0.44* (0.37-0.51) | 0.32* (0.27-0.38) |
| Retired | 0.32* (0.28-0.36) | 0.75* (0.66-0.85) | 0.42* (0.36-0.48) | 0.24* (0.20-0.28) | 0.63* (0.49-0.80) | 0.57* (0.45-0.73) |
| Family care | 0.47* (0.41-0.54) | 0.58* (0.52-0.64) | 0.65* (0.54-0.79) | 0.43* (0.36-0.52) | 0.53* (0.45-0.63) | 0.38* (0.32-0.45) |
| Full-time student | 1.26 (0.97-1.64) | 0.89* (0.79-0.99) | 0.71 (0.46-1.09) | 0.83 (0.55-1.26) | 0.60* (0.51-0.71) | 0.54* (0.45-0.65) |
| Other | 0.07* (0.06-0.08) | 0.25* (0.23-0.27) | 0.15* (0.13-0.17) | 0.03* (0.03-0.04) | 0.25* (0.22-0.29) | 0.09* (0.07-0.10) |

^a^ Variables adjusted mutually for each other.

*P-value<0.05.


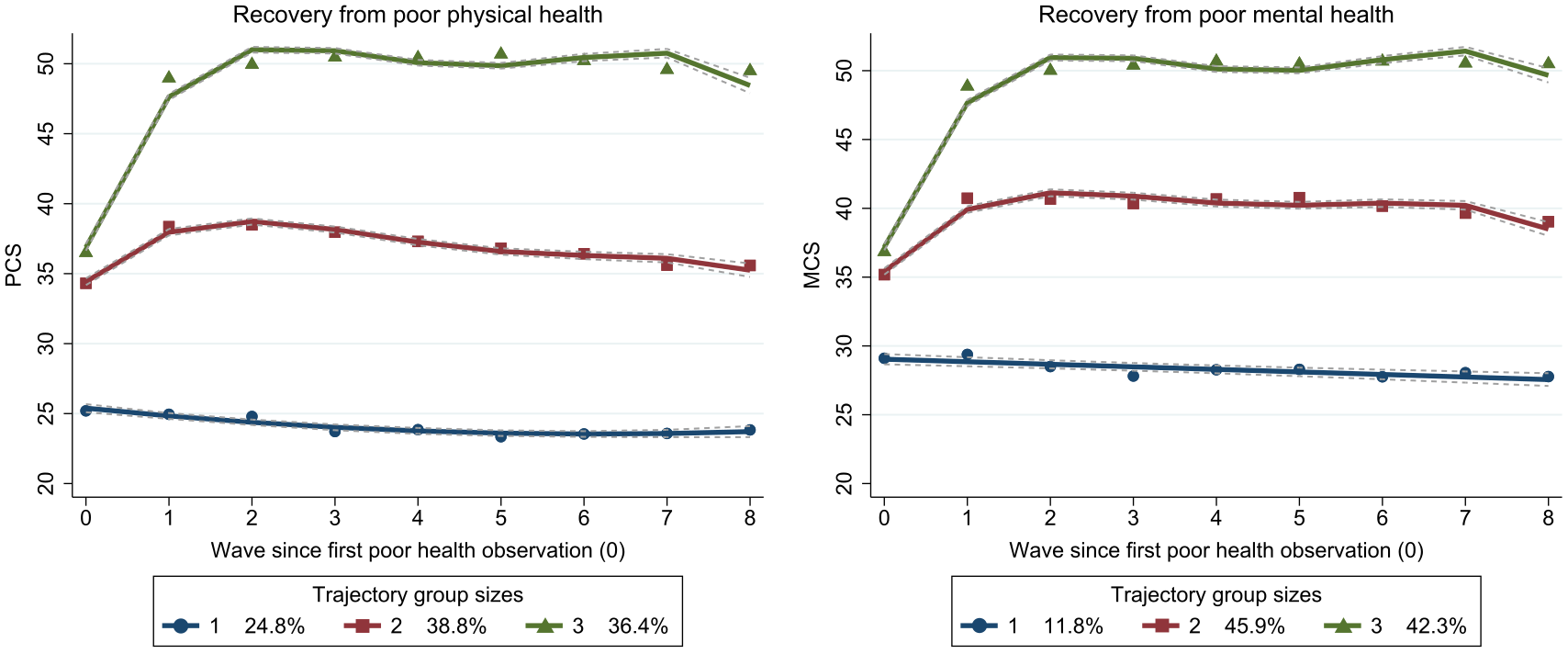


**Figure S1.** Physical and mental health recovery trajectories since participants’ first poor health observation, among those who had health functioning data at least in 5/9 Waves (N=10,406 for physical health and N=12,322 for mental health). Trajectory groups: 1=low-stable, 2=moderate-stable, 3=fast-increasing. Poor health was considered as belonging to the lowest health functioning quantile in the initial analytical sample (N=56,118). Physical and mental health functioning were estimated using physical and mental health component summary (PCS and MCS) scores from the 12-Item Short Form Survey (SF-12).
